# Evaluation of a Novel Methacrylate-Based Protein A Resin for the Purification of Immunoglobulins and Fc-Fusion Proteins

**DOI:** 10.1002/btpr.1951

**Published:** 2014-07-24

**Authors:** Tyler R McCaw, Edward K Koepf, Lynn Conley

**Affiliations:** Process Biochemistry, Biogen Idec, Research Triangle ParkNC, 27709

**Keywords:** Protein A chromatography, binding capacities, pressure–flow profiles, alkaline stability, mAbs, Fc-fusion proteins

## Abstract

Protein A affinity chromatography is a central part of most commercial monoclonal antibody and Fc-fusion protein purification processes. In the last couple years an increasing number of new Protein A technologies have emerged. One of these new Protein A technologies consists of a novel, alkaline-tolerant, Protein A ligand coupled to a macroporous polymethacrylate base matrix that has been optimized for immunoglobulin (Ig) G capture. The resin is interesting from a technology perspective because the particle size and pore distribution of the base beads are reported to have been optimized for high IgG binding and fast mass transfer, while the Protein A ligand has been engineered for enhanced alkaline tolerance. This resin was subjected to a number of technical studies including evaluating dynamic and static binding capacities, alkaline stability, Protein A leachate propensity, impurity clearance, and pressure–flow behavior. The results demonstrated similar static binding capacities as those achieved with industry standard agarose Protein A resins, but marginally lower dynamic binding capacities. Removal of impurities from the process stream, particularly host cell proteins, was molecule dependent, but in most instances matched the performance of the agarose resins. This resin was stable in 0.1 M NaOH for at least 100 h with little loss in binding capacity, with Protein A ligand leakage levels comparable to values for the agarose resins. Pressure–flow experiments in lab-scale chromatography columns demonstrated minimal resin compression at typical manufacturing flow rates. Prediction of resin compression in manufacturing scale columns did not suggest any pressure limitations upon scale up. © 2014 American Institute of Chemical Engineers *Biotechnol. Prog*., 30:1125–1136, 2014

## Introduction

Affinity chromatography employing immobilized Protein A is a well-established technology that is used extensively for industrial scale monoclonal antibody and Fc-fusion protein purification.^1^–^5^ Due to the strong binding interaction between the Fc moiety and Protein A, high purity is achieved in a relatively simple purification step. Clarified cell culture fluid containing the target protein of interest is applied to Protein A resin at near neutral pH, after which one or more wash steps are employed in order to reduce the levels of product and process related impurities such as host cell proteins (HCPs).^6^ Product desorption from the resin is achieved by a reduction of the mobile phase pH. Finally, the resin is regenerated and then subjected to a clean-in-place protocol. Due to the simple nature of this bind-and-elute operation, Protein A resins are very amenable for use in a platform process.^7^,^8^

Several commercially available Protein A affinity sorbents have been the subject of extensive studies, with performance comparisons published in a number of articles.^9^–^15^ Performance parameters that were studied included an assessment of equilibrium binding capacity, dynamic binding capacity (DBC) as a function of residence times, mass transfer properties, volumetric productivity rates, selectivity, and Protein A ligand stability. An ideal Protein A resin achieves high dynamic binding capacities, yields, and selectivity, demonstrates favorable pressure–flow properties, and is stable in commonly used cleaning agents for extended time periods.^16^ Two widely used agarose-based Protein A resins that achieve a balance between good binding capacity and throughput are MabSelect and MabSelect SuRe from GE Healthcare, the latter which has been designed for increased ligand stability in alkaline solutions.^9^,^17^

Recent changes in the Protein A intellectual property landscape have resulted in the emergence of new Protein A technology, both with respect to the design of the Protein ligand and the type of base matrix to which it is attached. A recent addition to the Protein A chromatography market is Amsphere Protein A JWT203 (JSR Life Sciences), which is comprised of a new, alkaline-stable, Protein A ligand immobilized on a novel methacrylate base matrix that has a mean particle diameter of 50 μm and narrow size distribution. The surface area of the resin is reported to be large at greater than 100 m^2^/g of resin beads, while the pore sizes vary from 10 nm to 5,000 nm. The polymer is synthesized from methacrylic monomers and organic solvents known as porogens, and then chemically modified to increase hydrophilicity in order to minimize nonspecific binding. The alkaline-stable ligand is attached to the base matrix via multi-point amine linkages. An ongoing endeavor at Biogen Idec is to assess and evaluate new purification technologies, and as such Amsphere Protein A resin was selected as a potential interesting candidate for further characterization.

The objective of this study was to evaluate Amsphere Protein A resin and compare its performance to the agarose-based resins MabSelect or MabSelect SuRe. The first part of the evaluation concentrated on binding capacity, both dynamic and static, using a select set of proteins being developed internally for therapeutic applications. Included in the set of molecules were three monoclonal immunoglobulin (Ig) G1s, designated Molecules A, B, and D, and one Fc-fusion protein, Molecule C. Selectivity studies followed the binding capacity work, which also included some Wash II screening studies in an effort to find solution additives capable of lowering contaminating HCP levels. Alkaline stability, Protein A ligand leakage, and pressure–flow properties completed the technical evaluation.

## Materials and Methods

### Instrumentation

All chromatography experiments were conducted using either an AKTA Explorer 100 or AKTA Pilot purification system (GE Healthcare Life Sciences, Piscataway, NJ). Chromatography resins were packed in 0.66 cm ID Omnifit (Diba Industries, Danbury, CT) or GE XK 16, 26, and 50 columns using 1 M NaCl for the packing solution. The pressure–flow experiments with the XK 16 column were measured using an AKTA Explorer 100, while the AKTA pilot was used for the XK 26 and 50 pressure–flow studies. Protein concentrations were determined by absorbance measurements at 280 nm (A_280_) using a Perkin Elmer Lambda 25 UV/Vis (Waltham, MA) or C Technologies Solo VPE Slope (Bridgewater, NJ) spectrophotometer.

### Protein A resins

Amsphere Protein A JWT203 resin was supplied at no cost by JSR Life Sciences (Sunnyvale, CA), while MabSelect and MabSelect SuRe resins were purchased from GE Healthcare Life Sciences (Piscataway, NJ).

### Protein solutions

Cell culture fluid containing each molecule was produced in pilot scale fed-batch bioreactors and clarified via centrifugation and depth filtration at Biogen Idec. The mAbs used in the study were selected in part to cover a variety of isoelectric points and cell culture titers, while the Fc fusion protein was selected as a challenging molecule for purification due to its lower binding capacity and propensity to aggregate (Table1). Molecules A, B, and D are monoclonal antibodies (mAbs) of subclass IgG1, while Molecule C is an Fc fusion protein. Harvested cell culture fluid (HCCF) was applied to the different Protein A resins and purified using conditions developed previously for each test molecule. Protein G titer assays were used to determine protein concentrations in the HCCF. The purification process for Molecule A was developed a number of years ago with MabSelect resin, the state-of-the art Protein A resin available at that time, while Molecules B, C, and D were developed with the newer, alkaline-stable, MabSelect SuRe resin. The capture step for Molecule A is conducted at 2–8°C, while the rest of the molecules are captured at ambient temperature. Testing with Molecule D was limited to selectivity and Wash II solution screening studies. For the DBC experiments purified protein was diluted to cell culture expression titers and pH adjusted to typical Protein A load conditions.

**Table 1:** Select Properties of Molecules used in the Resin Evaluation Studies

Molecule	Type	Production Host	Isoelectric Point	Titer (mg/mL)*	pH*	Temperature*	Load Density†
A	IgG1	NS0	8.9	0.6	6.5	2–8°C	35
B	IgG1	CHO	6.6	3.8	7.3	Ambient	35
C	Fc-Fusion	CHO	7.4	2.1	7.3	Ambient	20
D	IgG1	CHO	7.2	4.1	7.3	Ambient	35

* Protein A column load condition.

† Column load density (mg of protein loaded per mL of resin).

### Protein G titer assay

Protein titer assays were run on a Waters e2695 HPLC system equipped with a Waters 2998 Photodiode Array detector and a 2.1 mm × 30 mm POROS G20 affinity column (Life Technologies, Grand Island, NY). The assay was run at ambient temperature with the samples held at 2–8°C in the auto sampler until injection. Absorbance was monitored at 280 nm. The column was equilibrated in binding buffer (25 mM sodium phosphate, 200 mM NaCl, pH 6.5) at a flow rate of 2 mL/min, followed by sample injection. After sample application the column was washed for 3.5 min with binding buffer, followed for 3.5 min with the elution solution (200 mM NaCl, pH 2.0). The mass of bound protein (μg) was derived by integration of the eluate peak area and interpolation using a standard curve, while the titer reported in units of mg/mL was calculated by dividing the amount of bound protein by the sample injection volume.

### Dynamic binding capacity

Purified protein was loaded onto each 0.66 cm ID × 10 cm bed height column to the point at which the column effluent A_280_ exceeded the 10% load absorbance threshold. Prior to a run load material was passed through the flow cell of the instrument in order to establish the 100% absorbance signal. Dynamic binding capacity (DBC) was measured at several linear velocities corresponding to residence times spanning approximately 0.5 min to 6.0 min for molecules A, B, and D. Due to lower binding affinity of Fc fusions to Protein A, the residence time for molecule C was extended out to 15.6 min. The 10% breakthrough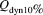 (mg of protein bound per mL of resin) was plotted as a function of residence time and then analyzed by the non-linear relationship according to Eq. 1. In this relationship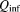 is the binding capacity at infinite residence time,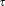 is the residence time, and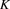 is fitting parameter with no physical significance.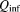 and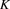 were determined using the Solver function in Microsoft Excel.


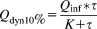
(1)

### Static binding capacity

Equilibrium binding isotherms were generated by batch uptake experiments. Aliquots containing a fixed amount of resin were incubated with varying amounts of purified protein in equilibration buffer. 2 mL vials were charged with 1 mL aliquots of well-mixed resin slurry such that an estimated 0.25 mL of settled resin was transferred to each vial. The transferred volume was divided by a factor of 1.1, reducing the resin volume slightly to reflect compression in a packed bed. A dilution series of each test molecule was prepared in equilibration buffer and 1 mL aliquots were added to the vials. The vials were agitated gently for 48 h on a rotating platform to achieve binding equilibrium. At the completion of the incubation period the resin was compacted using a Sorvall Legend 14 bench top centrifuge operated at 8,050*g* for 5 min. The protein concentration in the decanted supernatant was quantified by A_280_ measurements. Binding capacity (mg of bound protein per mL of resin) was plotted as a function of supernatant protein concentration to yield the Langmuir binding isotherm. Experimental data were fit to Eq. 2, where *Q*_max_ is the equilibrium binding capacity, *q* and *c* are the respective stationary and mobile phase protein concentrations, and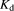 is the equilibrium dissociation constant.^18^


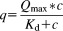
(2)

### Selectivity evaluation

HCCF was loaded to 0.66 cm ID × 10 cm bed height columns packed with virgin resin at the operational load density specified for each molecule. Molecules A, B, and D, were loaded to a density of 35 mg/mL, while Molecule C was loaded to 20 mg/mL (Table1). Process specific operating conditions (load, wash, and elution steps) developed for each molecule on the agarose resins were replicated on JSR Protein A. Eluate protein concentrations were determined by A_280_, while aggregate and HCP levels were measured using standard in-house analytical assays. SEC-HPLC was used to monitor aggregate levels using a Tosoh Biosciences TSKgel G3000SW analytical column (King of Prussia, PA), while HCPs were measured by ELISA with electrochemiluminescence detection based upon the Meso Scale Discovery platform (Rockville, MD). Residual Protein A (Pro A) leachate levels were determined with an in-house ELISA. Recombinant protein A was used to standardize the ELISA for the MabSelect and Amsphere runs, while the MabSelect SuRe ligand was used as the ELISA standard for molecules purified with MabSelect SuRe resin. When run in our ELISA format, the concentration of a known sample of pure JSR ligand was under-estimated by 45%, thus all JSR ligand leachate concentrations reported herein have been corrected accordingly.

### Wash II solution screening studies

Molecules C and D were selected as candidates for Wash II solution screening studies. These experiments were run in a manner as described in the selectivity evaluation section above, with the noted exception that three column volumes (CVs) of a unique Wash II buffer solution were employed for each experiment. Wash II solution formulations investigated included a high and low pH control (25 mM Tris–HCl, pH 9.0 or 25 mM sodium citrate, pH 4.4), control plus 1 M urea, control plus 10% isopropyl alcohol (IPA) by volume, and control plus 1 M urea and 10% IPA.^16^ In addition, the zwitterionic surfactant lauryldimethylamine oxide (LDAO) was evaluated at 10 and 20 mM. Each formulation was tested at the high (9.0) and low (4.4) pH conditions. Eluate pools were collected and analyzed for HCP content.

### Alkaline stability

Virgin Amsphere Protein A resin packed in 0.66 cm ID × 10 cm bed height columns was exposed to a pair of high pH cleaning solutions for extended time periods. At regular intervals during the alkaline solution contact experiment the resin was taken out of the high pH environment and tested for DBC using Molecule B. In Experiment 1 resin was exposed to 20 mM sodium phosphate, 1 M NaCl, and 11 mM NaOH at pH 11.2 for a contact time equivalent to 200 cycles (24 min per cycle), followed by an additional 40 cycles of harsher alkaline solution of 0.1 M NaOH (30 min per cycle). Experiment 2 entailed exposing resin to a contact time equivalent to 200 cycles (30 min per cycle) with only the 0.1 M NaOH solution. Eluates from select DBC cycles were assayed for leached Protein A.

### Pressure–flow experimentation

Columns of various aspect ratios containing virgin Amsphere Protein A resin were packed in 1 M NaCl to the critical bed height starting with a gravity-settled bed. As pressure–flow data for the agarose resins were available in the literature, only the Amsphere Protein A resin was evaluated for pressure–flow in this study. GE Healthcare Life Sciences XK 16, XK 26, and XK 50 columns were tested at target bed heights of 10 cm and 20 cm, generating data at six aspect ratios (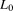/D), where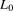 is the gravity-settled bed height and D the column inner diameter. Flow in each column was started slowly and then increased incrementally to a preset flow rate. The highest flow rates evaluated were 50, 165, and 250 mL/min for the XK 16, XK 26, and XK 50 columns, respectively. Flow rate, bed height, and pressure drop were measured once resin compression ceased and the bed height reached equilibrium at each incremental flow rate. Prior to testing the next flow rate the pump was stopped and the top flow adapter was lowered to a point just above the newly established bed height, displacing the buffer head that was generated due to resin compression. This procedure was to be repeated until small increases in flow were to result in substantial pressure drops.^19^ However, prior to achieving that state the experiments had to be terminated due to the pressure limitations of the columns. The XK 16 and XK 26 columns are rated to a maximum pressure of 0.5 MPa, while the XK 50 columns are rated up to 0.3 MPa.

### Pressure–flow data analysis

The experimental data from the XK 16, XK 26, and KX 50 pressure-flow experiments was used to determine the critical compression factor (λ_cri_) and the critical velocity (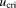) of each column aspect ratio tested. The experimentally derived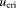 of each packing curve was then plotted in the form of a gravity-settled bed height normalized critical velocity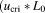) as a function of aspect ratio (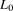 /D), which gives rise to the empirical linear relationship described by Eq. 3.^19^


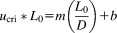
(3)

Once the above described linear relationship has been established with lab scale packing data it can be used to predict
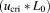
for any column aspect ratio, including large manufacturing columns were packing data with a new resin may be scarce or non-existent. The predicted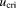 for a desired column aspect ratio is then used to calculate the compression factor (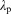) in that column at flow rate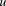 according to Eq. 4, where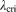 is derived from the lab scale pressure-flow experiments.


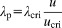
(4)

Knowing the compression factor (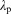) at flow rate (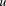) allows for the calculation of interstitial bed porosity (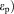 in that column under those flow conditions with Eq. 5, where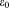 is the gravity-settled bed porosity.


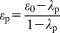
(5)

The gravity-settled bed porosity (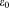) of 0.37 for the Amsphere Protein A resin was provided by the vendor, calculated from an experimentally determined interstitial bed porosity (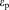) measured in a 1.0 cm ID column packed to low compression. The interstitial bed porosity
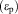
was derived by measuring the excluded bed volume using injections of blue dextran in a mobile phase of 50 mM sodium phosphate, and 7 M urea, pH 7.6. Correcting the measured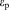 to account for compression produces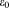.

Pressure-flow predictions were made using the Blake-Kozeny equation under the assumption of both incompressible and compressible resin behavior (Eq. 6). Here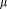 is the mobile phase viscosity,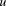 is the linear flow rate,
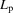
is the packed bed height,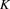 is a fitting parameter (commonly assumed to be 150),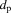 is the mean particle diameter, and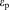 is the interstitial bed porosity.^19^ For the incompressible model calculations the following inputs were used:
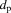
*=* 50 μm, K = 150, and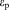 = 0.37. The compressible model used the same inputs as did the incompressible model, with the important distinction that a unique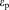 was calculated at each flow rate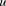 according to Eq. 5 noted above.


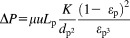
(6)

### Elution buffer pH and eluate pool volumes

Molecule A HCCF was loaded to a column packed with virgin Amsphere resin at 25 mg/mL load density and eluted with 10 mM sodium citrate buffer prepared at pH 3.2, 3.5 (control), 3.8, and 4.0. Eluate pool volumes were recorded and compared to those generated with MabSelect resin run at pH 3.5. A UV gate of 100–100 mAU was used to collect the eluate (2 mm path length).

## Results and Discussion

### Dynamic binding capacity

One of the most import performance attributes used to compare Protein A resins is DBC. This parameter is used to determine column sizing, which in turn defines the cycling strategy and processing time for the capture step at commercial scale.^13^ Hence, for a new Protein A resin to be considered for implementation into a purification process it must possess similar, if not superior, dynamic binding capacities as achieved with industry leading technology. The residence times evaluated as part of this study were not extended beyond those currently used in the capture step of each molecule in order to not change established cycle times.

**Figure 1:**
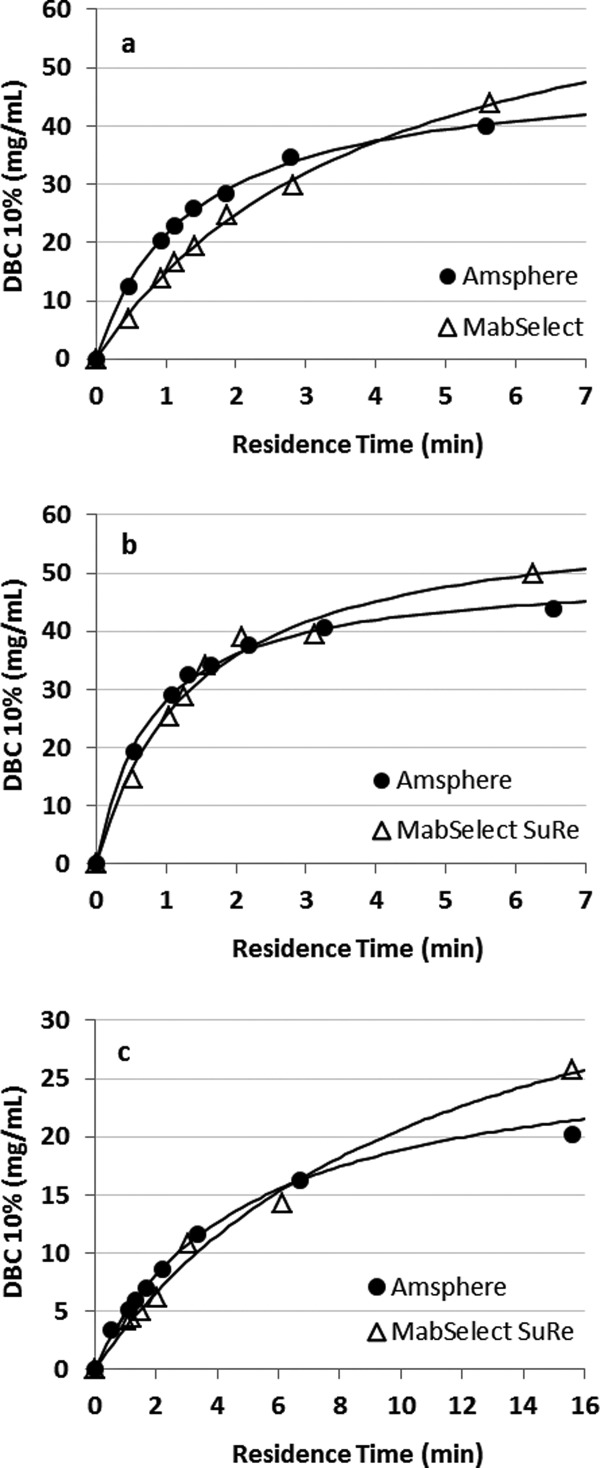
Dynamic binding capacity at 10% breakthrough as a function of residence time for (a) Molecule A, (b) Molecule B, and (c) Molecule C. The solid lines represent the best fit to the data according to Eq. 1.

The DBC reported at 10% breakthrough (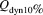) was measured for Molecules A, B, and C on both JSR and GE Protein A resins at several residence times (Figure 1). The plot of DBC as a function of residence time for all three molecules produced a logarithmic profile, illustrating increased binding capacities at longer residence times on all resins. At a residence time of 5.6 min and 2–8°C (process specific operating conditions) the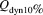 for Molecule A on Amsphere was 40 mg/mL, while GE MabSelect achieved a slightly higher capacity of 44 mg/mL. At ambient temperature and at a residence time of 6 min Molecule B achieved binding capacities of 45 mg/mL and 50 mg/mL on Amsphere and MabSelect SuRe resin, respectively.

As expected, the DBC achieved with the Fc-fusion protein (Molecule C) was much lower than what was observed with the mAbs on both Amsphere and MabSelect SuRe. Using the process specific operating conditions developed specifically for this molecule (15.6 min residence time and ambient temperature) the DBC on Amsphere just exceeded 20 mg/mL, while the agarose control resin achieved a higher DBC at nearly 26 mg/mL. The lower DBCs with Fc-fusion proteins are likely a consequence of steric interference manifested by the extended, flexible geometry of the Fc scaffold which may block access to adjacent binding sites.^20^

For each of the three molecules tested the agarose-based resins performed slightly better by margins of 9%, 10%, and 23% for Molecules A, B, and C, respectively, at process specific residence times, while at residence times of less than 3 min Amsphere performed similarly, if not better, than the agarose resins. DBCs are largely governed by particle size, pore size, and ligand density.^11^ Since the mean particle size for Amsphere (50 μm) is smaller than for the agarose resins (85 μm), one may surmise more efficient mass transfer and thus higher dynamic binding capacities with this resin assuming comparable molecular diffusivities. Given that the smaller particle size resin with a large surface area did not show superior DBC, pore size or ligand density differences between the resins may be the cause of the different binding capacities. Since the ligand density for the JSR resin is not known, the direct cause of the slightly lower DBCs is difficult to ascertain. However, given that the equilibrium binding capacities compare favorably between the resins (see section below), less efficient mass transport likely contributes in part to the slightly lower DBCs achieved with Amsphere. The run conditions that were used for this study had been optimized for the agarose resins, and as such may not have been ideal for Amsphere Protein A, which may have also contributed to the slightly lower DBCs.

### Static binding capacity

**Figure 2:**
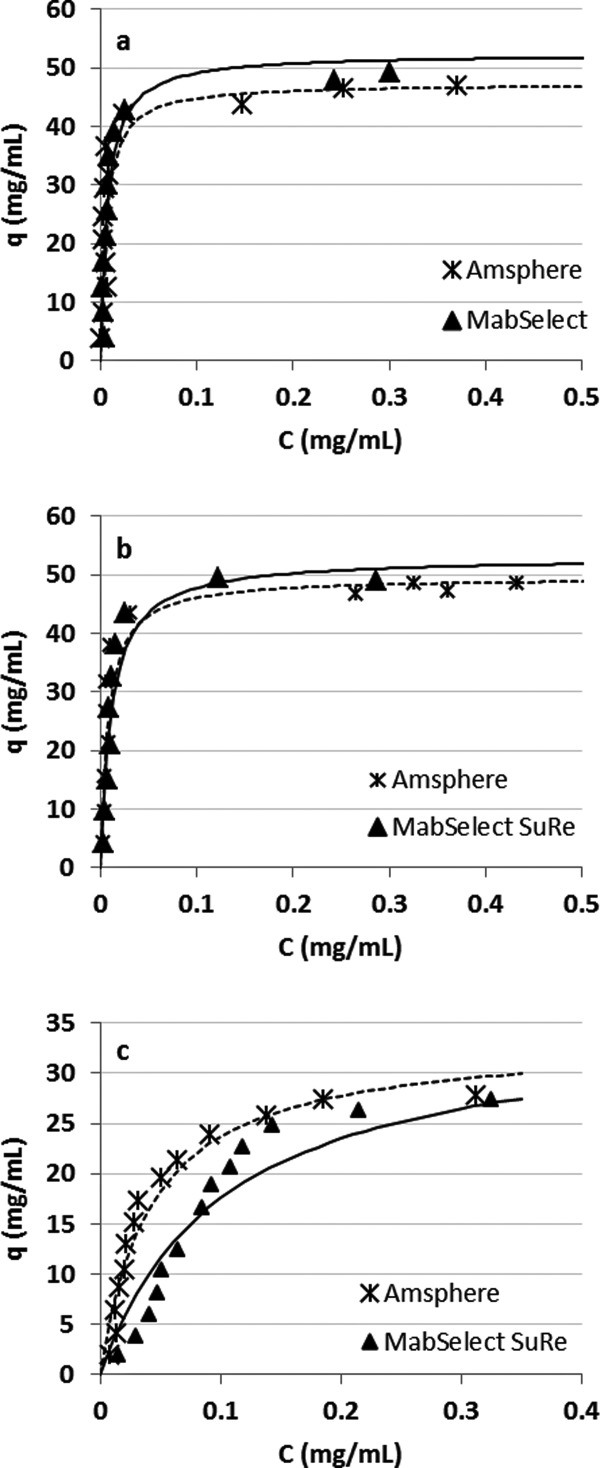
Static binding capacity (mg of adsorbed protein per mL of resin) as a function of unbound protein concentration after 48 h of incubation for (a) Molecule A, (b) Molecule B, and (c) Molecule C. The dashed (JSR) and solid (GE agarose) lines represent the Langmuir isotherms according to Eq. 2.

The equilibrium binding isotherms for Molecules A, B, and C on Amsphere and the agarose Protein A resins after 48 h of batch uptake are presented in Figure 2. The amount of protein bound per unit volume of resin is plotted as a function of the unbound protein concentration and fit to Eq. 2 to yield the Langmuir binding isotherms. For Molecules A and B the binding isotherms are nearly rectangular and highly favorable, typical for IgGs binding to Protein A affinity resins.^11^ The static or equilibrium binding capacity (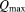) of Molecule A on Amsphere was 47.3 mg/mL, which compares well to the performance of MabSelect at 52.4 mg/mL (Table2). Similarly, for Molecule B the static binding capacities were 49.6 mg/mL and 52.9 mg/mL on Amsphere and MabSelect SuRe, respectively. The binding capacity of the Fc-fusion protein (Molecule C) was significantly less than those of the two mAbs. A binding capacity of approximately 28 mg/mL was measured for both Amsphere and MabSelect SuRe at the highest concentrations tested in the experiment, while the predicted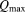 is 33.5 mg/mL and 35.5 mg/mL for Amsphere and MabSelect SuRe, respectively. Given that the binding isotherms for the Fc-fusion protein were relatively shallow and that the system does not yet appear to be in equilibrium, it may have been prudent to test higher concentrations of protein in the experiment. The equilibrium dissociation constant (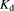) ranged between 39.2 nM and 74.8 nM for Molecules A and B, and it appears that the Protein A-ligand interaction was slightly stronger with the Amsphere resin. The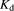 for the Fc-fusion protein was noticeably higher on both resins, with Amsphere again exhibiting an apparent stronger binding interaction. The static binding capacities measured with the two agarose resins are in the range of values reported in the literature.^11^,^20^ These results demonstrate similar equilibrium binding capacities between JSR and the GE agarose resins for the molecules that were evaluated as part of this study.

**Table 2:** Comparison of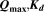,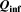, and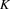 Between the Agarose and the JSR Protein A Resins

Parameter	Molecule A		Molecule B		Molecule C	
	MabSelect	Amsphere	SuRe	Amsphere	SuRe	Amsphere
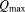 (mg/mL)	52.4	47.3	52.9	49.6	35.5	33.5
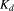 (nM)	47.0	39.2	74.8	52.6	955	386
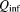 (mg/mL)	74.7	49.9	60.8	50.2	43.8	28.1
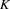	4.02	1.33	1.39	0.79	11.26	4.87

At infinite residence time the DBC should equal the static binding capacity. For the Amsphere Protein A resin there is general good agreement between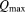 and empirically derived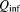 for all three molecules (Table2). This was not the case for the agarose resins, where for each molecule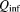 was considerably larger than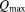. An examination of the DBC plots of Figure 1 shows that the curves for the agarose resins have not plateaued in the range of residence times tested, and as such fitting the data according to the empirical relationship described by Eq. 1 over-estimates the predicted DBCs when extended to very long residence times. DBC as a function of residence time data can be modeled using fundamental principles based upon mass transfer if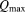 and the molecular diffusivity are known.^15^,^21^

### Selectivity evaluation

HCCF containing each test molecule was loaded to Amsphere and the agarose resins using process specific operating conditions. These conditions, which had been optimized for the agarose resins, were employed directly on Amsphere without any changes. Eluate pools from replicate runs (*n* = 2) were sampled and analyzed for high-molecular-weight (HMW) species, HCP, and Protein A leachate content (Table3). HCP reduction with Amsphere and the agarose resins was similar with Molecules A and B. For Molecule A, 2.9 logs of HCP clearance was achieved with both resins, while MabSelect SuRe performed marginally better than Amsphere with Molecule B (1.8 vs. 1.7 logs). HCP clearance was least effective with Molecule C on Amsphere resin. For this molecule, the log reduction achievable on Amsphere was only 1.4, whereas MabSelect SuRe cleared 1.9 logs. Molecule D showed the reverse trend in that the HCP clearance was more effective with Amsphere (2.4 logs) vs. MabSelect SuRe (2.0 logs). Due to the greater hydrophobicity of the polymeric methacrylate backbone of Amsphere, one may anticipate less effective HCP removal when compared to agarose-based resins.^22^ However, with the exception of Molecule C, the results of this study demonstrate largely comparable HCP clearance capabilities. The level of HMW species was similar in both the Amsphere and agarose eluates for all four molecules (Table3). Due to the complex structure of Molecule C a large percentage of that product from both resins aggregated, likely a consequence of exposure to the low pH environment of the eluate pool. Protein A leachate level comparisons between resins for all molecules are generally low and are in agreement with published values.^17^

**Table 3:** Impurity Clearance Performance of Amsphere and GE Agarose Resins

Assay	Molecule A		Molecule B		Molecule C		Molecule D	
	MabSelect	Amsphere	SuRe	Amsphere	SuRe	Amsphere	SuRe	Amsphere
Load HCP (ppm)	631,000		106,000		205,000		120,000	
Eluate HCP (ppm)	967 ± 407	895 ± 132	2,430 ± 6	3,430 ± 513	3,880 ± 143	11,900 ± 931	1,600 ± 491	578 ± 47
Log_10_ HCP reduction	2.9 ± 0.1	2.9 ± 0.1	1.8 ± 0.0	1.7 ± 0.0	1.9 ± 0.0	1.4 ± 0.0	2.0 ± 0.1	2.4 ± 0.0
HMW (%)	0.4 ± 0.0	0.5 ± 0.0	4.8 ± 0.4	5.3 ± 0.6	38.0 ± 0.9	36.6 ± 0.1	3.4 ± 0.4	4.0 ± 0.3
Pro A (ppm)*	4.2 ± 4.3	4.0 ± 2.7	5.6 ± 4.3	8.5 ± 4.0	9.7 ± 1.4	19.4 ± 15.6	7.8 ± 3.1	5.8 ± 0.0

* The Amsphere Pro A leachate values have been adjusted to account for approximately 45% less signal in the ELISA.

### Wash II solution screening experiments

Protein A affinity chromatography typically achieves very good clearance of HCPs and other process related impurities due to the high binding affinity of the Fc-domain of mAbs and Fc-fusion proteins to Protein A. The majority of the HCPs accumulate in the flow through pool, but some do coelute with the product which can then negatively impact downstream polishing steps.^16^ In order to maximize HCP clearance at the capture step various wash strategies have been investigated, including implementing a second wash step with a buffer that is at a pH intermediate to that of the load and elution buffer.^6^ The intent of a second wash buffer is to disrupt HCP-product and HCP-resin binding without diminishing the strength of the Protein A-product interaction.^16^ Since hydrophobic and electrostatic interactions mediate the largely nonspecific HCP binding, solution additives that are able to attenuate these forces are good candidates for use in a second wash step. Chaotropes, detergents, salts, and organic solvents, either alone or in combination, have all been used successfully as additives in a Wash II step.^6^

During the selectivity experiments the greatest variation in HCP clearance between Amsphere and the agarose resins was encountered with Molecules C and D. For Molecule C, MabSelect SuRe proved to be more effective, while the reverse was seen for Molecule D. In an attempt to lower the HCP levels in the eluates of these two molecules several solution additives were tested as part of a Wash II formulation screening study. The choice of which buffer conditions and additives to test was motivated in-part by a study from Shukla and Hinckley,^16^ who investigated a chaotrope, lowering the polarity of the mobile phase, the addition of a detergent, and combination strategies at high and low pHs. The components screened in our Wash II solution study are by no means exhaustive, but they do span many of the classes of additives commonly employed for Protein A chromatography.

The results of the Wash II solution screening experiment with Molecules C and D on Amsphere resin are presented in Table4. Upon data review, a trend was evident with Molecule C and the pH 9.0 Wash II solutions. Isopropanol (10% IPA) was marginally better at HCP reduction than 1 M urea, while the combination of both was more efficacious than either component alone. The urea/IPA combination was able to reduce the HCP concentration from 11,900 ppm (base process) to 3,710 ppm, similar to the level achieved with MabSelect SuRe at 3,880 ppm (Table3). LDAO at 20 mM provided some additional HCP clearance, reducing the eluate HCP concentration further to 2,870 ppm. These observations seems to indicate that Amsphere is not inherently less selective, but rather that this resin will require some level of optimization for certain feed streams. These Wash II solutions had less of an impact on HCP removal with Molecule D (Table4). Most effective HCP reduction was achieved again with the Wash II solution that contained both urea and IPA. The HCP reduction realized with LDAO and Molecule C did not transfer to Molecule D. The high pH solutions were consistently better at removing HCPs than their lower pH counterparts, particularly with Molecule D where none of the pH 4.4 solutions performed as well as the base process. These results demonstrate that the HCP levels can be reduced further by the informed selection of a Wash II step for some feed streams, but in order to do so product specific optimization will be necessary.

**Table 4:** Wash II Solution Screening Experiments for HCP Reduction on Amsphere Resin

	HCP (ppm)		
Wash II Solution Composition		Molecule C	Molecule D
Base Process		11,900	578
25 mM Tris–HCl, pH 9.0	pH 9.0	7,350	501
1 M urea		5,200	425
10% IPA		4,440	494
Urea/IPA		3,710	344
10 mM LDAO		5,140	572
20 mM LDAO		2,870	501
25 mM sodium citrate, pH 4.4	pH 4.4	12,220	904
1 M urea		8,330	1,100
10% IPA		6,670	1,050
Urea/IPA		4,640	793
10 mM LDAO		10,900	1,070
20 mM LDAO		8,860	753

### Alkaline stability

Due to the substantial cost of Protein A resins commercial scale Protein A capture columns are typically cycled several times per batch and reused for multiple batches. As the useable cycle count increases the cost of resin per cycle decreases, so maximizing resin lifetime is important to the overall process economics. Over the functional lifetime of the resin it has to exhibit robust purification performance and deliver reliable process consistency. Two well-known contributors to Protein A performance deterioration are irreversible binding of target protein or cell culture impurities which may lead to resin fouling, and hydrolysis of the Protein A ligand by contact with proteases or exposure to cleaning/sanitization solutions.^23^ To prevent fouling a number of diverse cleaning agents may be used, including high concentrations of urea, guanidine hydrochloride, reducing agents, acids, and alkaline solutions.^6^ Of these options NaOH is very desirable due to its cleaning and sanitization effectiveness, virus inactivation, endotoxin degradation, low cost, and nonspecialized waste disposal.^9^ However, the concentration of NaOH needed to prevent resin fouling is often detrimental to Protein A ligand stability, which negatively impacts resin lifetime. To overcome this limitation the Protein A ligand can be engineered for enhanced alkaline stability, such as the ligand that is part of MabSelect SuRe.^9^ The ligand on Amsphere Protein A has also been engineered for increased alkaline tolerance, and as such the resin was subjected to two NaOH solution exposure experiments; the first with a mild cleaning solution (20 mM sodium phosphate, 1 M NaCl, 11 mM NaOH, pH 11.2) and the second with a stronger sanitization solution (0.1 M NaOH). For these experiments, the resin was not actually cycled through a chromatography run, but instead exposed to the cleaning/sanitizing solutions for corresponding contact times. The first experiment was designed to emulate a hypothetical mAb purification process with a desired resin lifetime of 200 cycles, where each cycle would include exposure to the cleaning solution and then every fifth cycle to the harsher sanitization solution.

**Figure 3:**
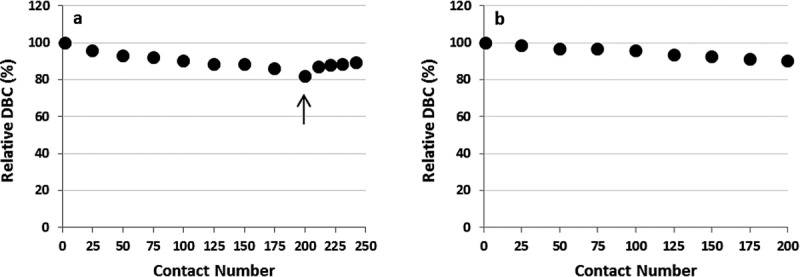
Normalized DBCs on Amsphere resin as a function of alkaline solution contacts at ambient temperature. (a) Exposure to 20 mM sodium phosphate, 1 M NaCl, 11 mM NaOH, pH 11.2 for 24 min per contact and (b) exposure to 0.1 M NaOH for 30 min per contact. The arrow in (a) demarks the point where the resin was exposed to the 0.1 M NaOH sanitization solution.

For the first alkaline solution contact experiment the initial DBC on virgin Amsphere resin was 45.4 mg/mL with Molecule B, run at a residence time of 6 min in a 0.66 cm ID × 10 cm bed height column. When exposed to the pH 11.2 cleaning solution the DBC decreased marginally over the first 50 exposures by approximately 7%, and then to 11.5% around 150 exposures (Figure 3a). A further small DBC decrease was observed between exposures 175 and 200. Contact with 0.1 M NaOH sanitization solution after exposure 200 restored the DBC to 40 mg/mL or 88% of the initial capacity, suggesting that resin fouling contributed in part to the decreased binding capacity. Confirmation of protein build-up on the resin could have been made with a standard resin extraction experiment.

To support the hypothesis that predominately inadequate cleaning and not ligand degradation led to the decrease in the binding capacity with the milder pH 11.2 cleaning solution a second alkaline solution exposure experiment was conducted, but this time using only the harsher 0.1 M NaOH sanitization solution (Figure 3b). In this experiment less than a 10% decrease in binding capacity was observed over the 200 exposures (100 h), suggesting that 0.1 M NaOH is an effective cleaning solution that does not adversely affect the stability of the Amsphere ligand for an exposure time of at least 100 h. MabSelect SuRe exposed to 0.1 M NaOH for 200 cycles at 15 min per cycle, which is equivalent to 100 cycles in this experiment (50 h), displays a DBC loss of approximately 10%, while the JSR resin lost about 5% of the initial DBC at that exposure time.^17^ This observation may indicate that the Amsphere ligand is slightly more alkaline-tolerant than the MabSelect SuRe ligand.

**Figure 4:**
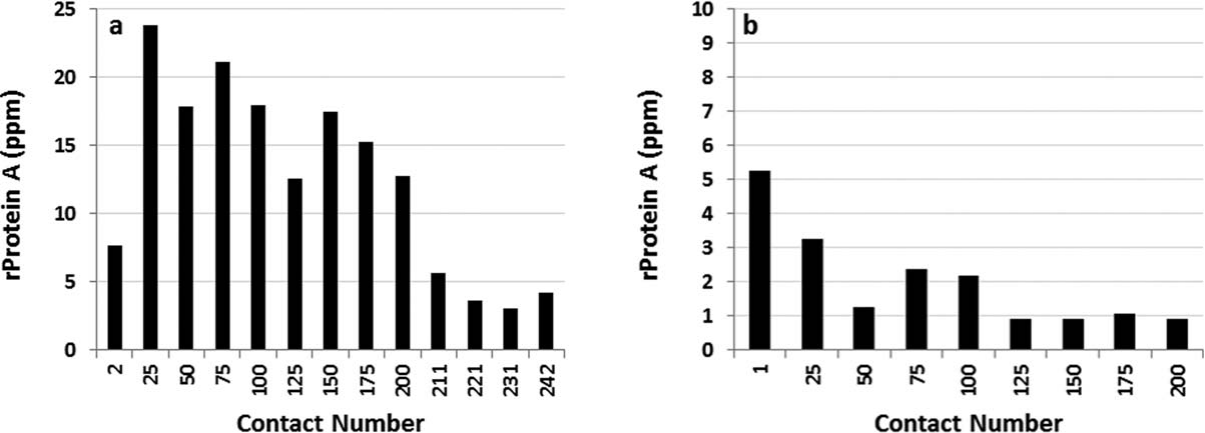
Amsphere ligand leakage monitored during the cleaning and sanitization solution exposure experiments. (a) leachate from exposure to 20 mM sodium phosphate, 1 M NaCl, 11 mM NaOH, pH 11.2 and (b) exposure to 0.1 M NaOH. The Pro A leachate values have been adjusted to account for approximately 45% less signal in the ELISA.

During select DBC runs the protein that was loaded to the column was eluted using a 100 mM glycine, pH 3.0 buffer and those eluates were then subjected to Protein A leachate analysis (Figure 4). For both experiments, ligand leaching was consistently low and at levels reported for MabSelect SuRe.^12^,^17^ As noted previously the ELISA that was used to measure Protein A leachate in the Amsphere samples was developed for the detection of standard recombinant Protein A ligand. In this assay the Amsphere ligand concentration is underestimated by approximately 45% (data not shown), hence the leachate data presented in Figure 4 have been adjusted to account for this difference.

The results from this alkaline solution contact study demonstrate that the resin is stable in the pH 11.2 cleaning solution for a contact time equivalent to 200 cycles (24 min/cycle), or in the 0.1 M NaOH sanitization solution for at least 200 cycles (30 min/cycle). Given the potential of resin fouling when only using the cleaning solution along with the demonstrated stability exhibited in 0.1 M NaOH, an effective yet simple cleaning strategy would be to just use 0.1 M NaOH for each product cycle. Additional data are needed to verify the stability beyond 200 sanitization solution contacts, ideally running a comprehensive cycling study using a more challenging feed stream such as HCCF.

### Pressure–flow profiles

Resins composed of rigid, polymeric, base matrices may be assumed to be largely incompressible at the low flow rates usually employed for large-scale manufacturing, even though some degree of compression does occur. Although a more accurate pressure-drop prediction is achieved if the interstitial bed porosity is recalculated at each flow rate tested due to resin compression, the calculations are simplified considerably if incompressibility is assumed. To determine which assumption is more appropriate for predicting pressure–flow profiles with the Amsphere Protein A resin, experimental packing data were fit to both a compressible and incompressible model.

The pressure–flow properties of the JSR Protein A resin were evaluated in lab-scale columns following the methodology described by Stickel and Fotopoulos.^19^ These data were used to generate a simplified empirical model useful for predicting pressure drops across columns of any aspect ratio (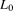/D), where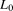 is the gravity-settled bed height and *D* is the column inner diameter. Being able to predict the pressure–flow properties of a resin in commercial scale columns early on in development is exceedingly beneficial as that information can be used to help define operating conditions suitable for implementation at commercial scale. This is particularly important for a new resin composed of a base matrix where scale-up packing data are not available. The column diameters and aspect ratios that were tested as part of the pressure–flow evaluation are listed in Table5. Due to limited resin availability the XK 50 column with a target bed height of 20 cm (aspect ratio of 4.00) could not tested.

**Table 5:** Column Dimensions and Aspect Ratios Tested for Pressure–Flow Evaluation

Diameter (cm)	Bed Height (cm)	Aspect Ratio
1.6	10	6.25
1.6	20	12.50
2.6	10	3.85
2.6	20	7.69
5.0	10	2.00

**Figure 5:**
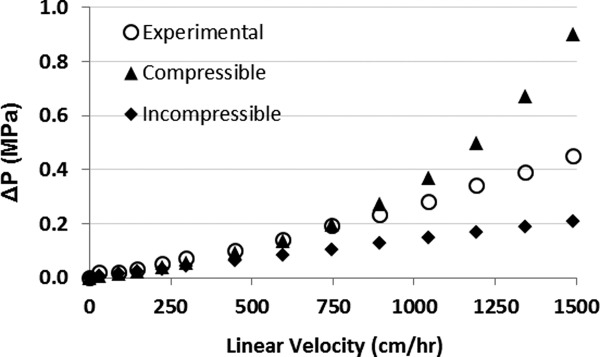
Experimental and predicted pressure–flow profile for a 1.6 cm *×* 10 cm bed height column packed with JSR Protein A resin. The open circles represent the experimental pressure-drop data, the solid triangles are predicted values calculated using the Blake–Kozeny equation with εp varying as a function of flow rate (compressible model), while the solid diamonds are predicted values assuming noncompression.

A 1 M NaCl solution was applied to each gravity-settled resin bed at progressively increasing linear velocities until a target flow rate had been reached. Once resin compression ceased, the incurred pressure drop, volumetric flow rate, and bed height were recorded. The flow adaptor was then repositioned to the top of the newly compressed bed and the process was repeated at several flow rates. The experimental pressure–flow data for the 1.6 cm ID × 10 cm bed height column are shown in Figure 5, as is the predicted profile derived using the Blake–Kozeny relationship (Eq. 6) assuming both compressible and incompressible behavior. For the compressible model the interstitial bed porosity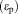 in the Blake–Kozeny equation varied as function of flow rate. It was calculated with Eq. 5, which uses the compression factor (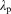) at each flow rate as an input. The compression factor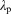 at each flow rate was, in turn, calculated using Eq. 4 with the experimentally derived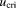 of 1159.3 cm/h and a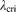 of 0.12. The flow rate and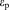 were the variable inputs into Eq. 6 for calculating the pressure drop. For the incompressible model predictions the calculation were simplified as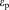 was assumed to be constant (0.37) and only flow rate varied.

The experimental data show that the relationship between pressure drop and superficial velocity is almost linear in the range of flow rates tested, suggesting that the JSR Protein A resin behaves more like a rigid, incompressible medium. The experimental and incompressible pressure drop predictions start to diverge around 500 cm/h, indicating the occurrence of some resin compression above this flow rate. At the high end of the flow rates tested (1,500 cm/h) the experimental pressure drop is over twofold higher than what is predicted assuming no compression, but approximately twofold lower than what is predicted by the compressible model.

### Prediction of pressure–flow in larger columns

**Figure 6:**
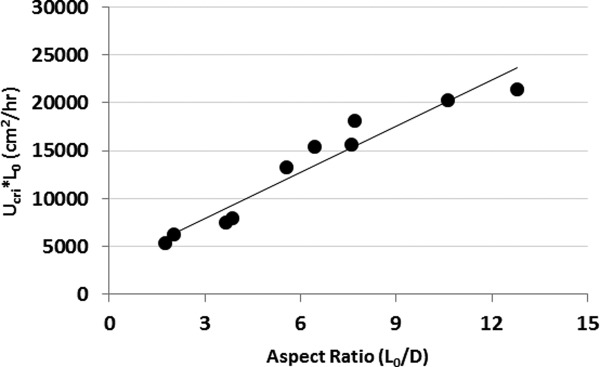
Empirical relationship of the critical velocity normalized to the gravity settled bed height with the column aspect ratio.

The pressure–flow experimental procedure detailed above was repeated for all the column aspect ratios listed in Table5. For each pressure–flow experiment the critical velocity (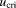) and critical compression factor (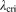) were determined. The average of the critical compression factors was 0.12, and varied little with column aspect ratio. Determination of the critical velocity in these experiments was somewhat subjective as column pressure ratings prevented flowing at velocities high enough such that the slope of the pressure–flow curve approached infinity.^19^ For these experiments the critical velocity was defined as the flow rate where the pressure–flow curve deviated from linearity. A plot of
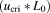
as a function of aspect ratio (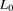 /D), as described by Eq. 3, for all columns is presented in Figure 6. Linear regression of the data produces the empirical relationship *y* = 1603.5*x* + 3160.6 (*r*^2^ = 0.9326). This relationship was used for predicting the pressure–flow profile of a 20 cm ID × 20 cm bed height column packed with Amsphere Protein A resin, where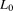 /D = 1. This specific column configuration was chosen for predicting pressure–flow as experimental packing data in such a column was available from the resin vendor. With this aspect ratio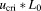 = 4764.1, a value which was then used to derive a critical velocity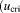) of 209.9 cm/h with a gravity-settled bed height (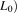 of 22.7 cm.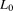 originated from adjusting the target packed bed height of 20 cm for compression using the critical compression factor of 0.12. Experimental packing data were compared to the pressure–flow predictions to gauge the accuracy of the models.

For the compressible model the pressure–flow predictions in the 20 cm × 20 cm column were derived using a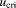 of 209.9 cm/h and 0.12 for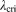 as noted above. These values were used as inputs in Eq. 4, which calculated a unique compression factor (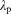) at each flow rate for which a prediction was to be made. Each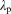 was then used an as an input to Eq. 5 to calculate the interstitial bed porosity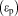. Finally, each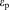 calculated at flow rate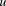 along with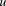 were inputs in the Blake–Kozeny equation for the pressure–flow predictions assuming resin compressibility. For the incomprehensible model the bed porosity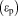 of 0.37 was used in Eq. 6, which was assumed to be constant at all flow rates.

**Figure 7:**
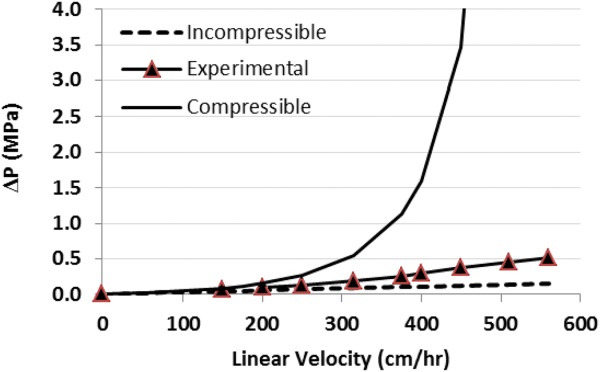
Comparison of pressure drop models derived assuming resin compressibility (solid line) and incompressibility (dashed line) to experimental data collected with a 20 cm ID × 20 cm bed height column (solid triangles). The experimental data were provided by JSR Life Sciences.

Analysis of the experimental packing data indicates that bed compression is not a significant concern up to approximately 500 cm/h in this column configuration given the pressure drop at this flow rate is still less than 0.5 MPa (Figure 7). At this flow rate the Amsphere Protein A resin shows some compression, with a pressure-drop that is slightly higher than what is predicted by the incompressible model. These larger scale packing data are consistent with the pressure–flow behavior achieved with the smaller XK 16 column, which also showed that the resin is only marginally compressible and that the pressure–flow profile is better predicted by the incompressible model (Figure 5). For comparison, published pressure–flow data with MabSelect SuRe resin with a bed height of 20 cm show pressure drops of approximately 0.1 MPa and 0.14 MPa in 10 cm and 30 cm diameter columns, respectively.^24^ Although the agarose-based MabSelect SuRe base matrix is softer than the more rigid polymeric methacrylate resin beads, the pressure–flow profile for the agarose resin is more favorable likely due to a larger mean particle diameter of 85 μm.

Pressure drops are not anticipated to be a concern when scaling up to typical large manufacturing columns (100 cm ID or larger × 20 cm beg height) based upon predictions made from this set of experimental data. However, as wall effects are still prevalent in packed beds with inverse aspect ratios (*D*/*L*_0_) less than 2, the small-scale experimental data used to make the pressure–flow predictions must still be interpreted with some caution given that the largest inverse aspect ratio evaluated in this experiment was only 0.5.^19^ Frictional forces between the wall of the column and resin particles provide some degree of support for the resin bed in small ID columns, facilitating higher superficial velocities.^25^ As such support deceases with increasing column diameter, the loss of the wall effect needs to be considered when scaling-up to large manufacturing columns in order to avoid excessive bed compression. This is particularly important if scale-up is predicated on maintaining column bed height and residence time.

### Elution profiles

**Figure 8:**
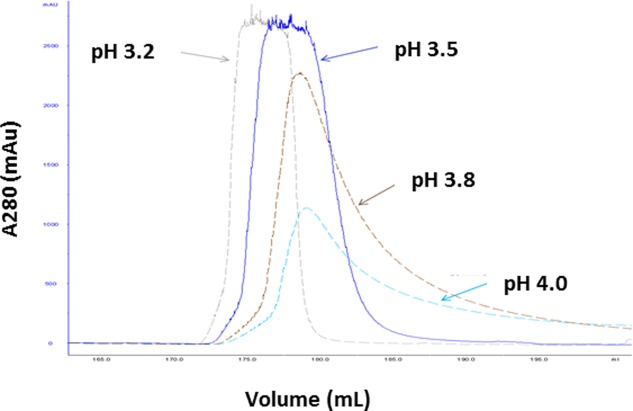
Effects of elution buffer pH on eluate peak shape and pool volume.

During Protein A chromatography development one of the performance parameters that is monitored is eluate pool volume, which is largely a function of elution buffer pH and column load density. The elution profile of Molecule A on Amsphere resin was monitored as a function of elution buffer pH. HCCF containing Molecule A was loaded to Amsphere Protein A to a density of 25 mg/mL, and then eluted with buffers of identical concentration but at different pHs. Presented in Figure 8 is an overlay of the elution peaks of each experimental run which were all collected using an identical UV collection gate of 100–100 mAU (2 mm path length).

As can be seen in Figure 8 the elution buffer pH had a profound effect on eluate pool volume. Molecule A was desorbed most effectively with lower pH buffers, while the higher pH buffers resulted in expanded eluate pool volumes due to significant tailing, congruous with the behavior of other Protein A resins. Peak collection ceased after 2.27, 3.62, 9.16, and 9.98 CVs had been collected with the pH 3.2, 3.5, 3.8, and 4.0 elution buffers, respectively. When run on MabSelect resin at pH 3.5 (process specific conditions) the eluate pool volume of Molecule A was considerably smaller at only 1.90 CVs. However, smaller eluate pool volumes were achieved on Amsphere resin with Molecule B and a lower pH elution buffer. Molecule B purified over Amsphere with a pH 3.0 elution buffer was collected in 1.59 CVs, which was lower than the volume of 2.00 CVs achieved with MabSelect SuRe. Collectively these results demonstrate that lower pH elution buffers are most effective for use on this resin, although the choice of which buffer to use is ultimately dictated by product stability.

## Conclusions

In this study, we have investigated the performance of Amsphere Protein A affinity resin, a recent addition to the growing repertoire of Protein A resins available for mAb and Fc-fusion protein purification, and compared its performance to industry leading agarose-based Protein A technology. Equilibrium binding capacities were comparable between Amsphere and the agarose resins (MabSelect and MabSelect SuRe) for all molecules tested. The binding capacities of the mAbs (Molecules A and B) on the JSR resin were high and within expectation, but marginally lower than what was achieved on the agarose resins, while near identical binding capacities were realized with the Fc-fusion protein (Molecule C). DBC experiments revealed somewhat larger performance disparities between the resins, which showed slightly better binding on the agarose resins, most notably at extended residence times. Since pore diffusion mass transfer is dependent on particle size, longer residence times are expected to result in higher binding with the larger agarose particles. Furthermore, performance differences may be due in part to the fact that the operational processes used for the evaluations were developed and optimized with the agarose resins. These operating conditions were employed directly on Amsphere resin without any optimization. Amsphere was able to remove process related impurities (HCPs) to levels comparable with the agarose resins with Molecules A and B, surpassed the performance of MabSelect SuRe with Molecule D, but was not as efficient as MabSelect SuRe with Molecule C. However, through a brief screen of potential Wash II solution additives the combination of urea and isopropanol or the detergent LDAO at 20 mM was able to reduce copurifying HCPs to levels achieved with the agarose resin. Stability in 0.1 M NaOH for a contact time equivalent to 100 h was demonstrated in two separate alkaline solution contact experiments by virtue of achieving relatively constant DBCs and overall low rates of Protein A ligand leakage. Finally, the pressure–flow studies with Amsphere showed that the resin was slightly compressible, but pressure drop is nonetheless best estimated by an incompressible model. Moreover, scale-up to manufacturing size chromatography columns appears to be feasible without encountering pressure limitations when operating at typical commercial scale flow rates.

Results presented herein demonstrate that the Amsphere Protein A adsorbent performs similarly to the well-established agarose-based Protein A resins that are used extensively in the biopharmaceutical industry. Based upon largely similar static and dynamic binding capacities between the JSR and agarose resins, it may be feasible to substitute one resin for the other in a preparative process without changing column size, cycling strategy, and flow rate. Furthermore, ligand stability seems to indicate that resin lifetime will be comparable to the agarose resins. What remains to be determined is the performance of the resin when challenged with actual HCCF in a prolonged cycling study focusing on resin cleanability and batch-to-batch carryover, selectivity performance with a larger set of molecules, stability of the methacrylate base matrix in sanitization solution, the ease of packing a commercial scale column, and at scale pressure-drop performance.

## Greek Letters

=the gravity-settled bed porosity
=interstitial bed porosity
=critical compression factor
=compression factor
=mobile phase viscosity (Pa/s)
=residence time (min)

## Abbreviations

=mobile phase protein concentration (mg/mL)
*D*: =column diameter (m)
=mean particle diameter (m)
=equilibrium dissociation constant (nM)
=packed bed height (m)
=gravity-settled bed height (m)
=stationary phase protein concentration (mg/mL)
=dynamic binding capacity at 10% breakthrough (mg/mL)
=binding capacity at infinite residence time (mg/mL)
=equilibrium binding capacity (mg/mL)
=superficial velocity (m/s)
=critical velocity (m/s)
